# Ages of the Poets of America

**Published:** 1855-01

**Authors:** 


### AGES OF THE POETS OF AMERICA.

| | AGES. | | AGES. |
|---|---|---|---|
| James K. Paulding, - | 75 | Park Benjamin, | 45 |
| John Pierpont, - | 69 | James Freeman Clarke, | 44 |
| Richard H. Dana, - | 67 | Ralph Hoyt, - | 44 |
| Charles Sprague, - | 63 | James Aldrich, - | 44 |
| John Neal, - - | 60 | William H. C. Hosmer, | 44 |
| Willian C. Bryant, - | 60 | Jones Very, - | 44 |
| James G. Percival, - | 59 | Alfred B. Street, - | 43 |
| Fitz Greene Halleck, - | 59 | George W. Cutter, - | 43 |
| Samuel G. Goodrich, - | 58 | Willian H. Burleigh, - | 42 |
| George W. Doane, - | 55 | Henry T. Tuckerman, - | 41 |
| George P. Morris, - | 53 | Henry B. Hirst, - | 41 |
| Albert G. Greene, - | 52 | Cornelius Matthews, - | 39 |
| George W. Bethune, - | 52 | John G. Saxe, - | 38 |
| Ralph Waldo Emerson, - | 51 | Philip P. Cooke, - | 38 |
| George D. Prentice, - | 50 | Epes Sargent, - | 38 |
| Charles F. Hoffman, - | 48 | Thomas W. Parsons, - | 37 |
| N. P. Willis, - | 47 | George W. Dewey, - | 36 |
| William G. Simms, - | 47 | Arthur C. Coxe, - | 36 |
| Henry W. Longfellow, - | 47 | James T. Fields, - | 36 |
| George Lunt, - | 47 | James Russel Lowell, - | 35 |
| John G. Whittier, - | 46 | Thomas Buchanan Reed, | 32 |
| William D. Gallagher, - | 46 | George H. Baker, - | 31 |
| Oliver Wendell Holmes, | 45 | Bayard Taylor, - | 29 |
| Albert Pike, - | 45 | R. H. Stoddard, - | 28 |

*Boston Transcript.*

